# Maternal supplementation of functional fiber improves reproduction performance by modulating gut microbiota during pregnancy

**DOI:** 10.3389/fmicb.2026.1758091

**Published:** 2026-02-24

**Authors:** Shengnan Yin, Jinghua Cheng, Mu Wang, Yuanfei Zhou, Hongkui Wei, Siwen Jiang, Jian Peng

**Affiliations:** 1 College of Animal Science and Technology, Huazhong Agricultural University, Wuhan, China; 2 Tecon Biology Co., Ltd., Urumqi, China; 3 Frontiers Science Center for Animal Breeding and Sustainable Production, Wuhan, China; 4 The Cooperative Innovation Center for Sustainable Pig Production, Wuhan, China

**Keywords:** dietary fiber, fecal metabolites, gut microbiota, reproductive performance, sow

## Abstract

**Introduction:**

This study aimed to investigate the impacts of gestation diets supplemented with functional fiber on performance and gut microbiome of sows.

**Methods:**

A total of 1,000 healthy sows of comparable body weight (DanBred Landrace × DanBred Yorkshire, parities 1–2) were selected and randomly assigned to two dietary treatment groups after artificial insemination: a control group (CON, composed of beet pulp and barley as fiber sources) and a dietary fiber group [DF, supplemented with 1% functional fiber, consisted of 85.7% resistant starch (Hangzhou, China) and 14.3% guar gum (Yunzhou, China)].

**Results:**

DF treatment increased the numbers of total born, healthy piglets and litter birth weight (*p* < 0.05), whereas markedly decreased (*p* < 0.05) the number of intrauterine growth retardation (IUGR) compared with the CON group. Gut microbiota compositions underwent significant changes across gestation stages. Gut microbial diversity in DF group exhibited enhanced stability and resilience. Co-occurrence network analysis further demonstrated that the DF group maintained higher network stability at both G30 d and G109 d, with topological parameters consistently supporting these findings. In addition, *Treponema* showed a significant increase in the CON group starting from G30 d and persisted into late pregnancy (*p* < 0.05), whereas *NK4A214_group* showed a significant increase in the DF group at G30 d, G109 d and L14 d (*p* < 0.05). The abundance of *Treponema* was negatively correlated with the numbers of total born (*p <* 0.01) and healthy piglets (*p* < 0.05). *NK4A214_group* showed a positive correlated with the numbers of total born and born alive (*p* < 0.05), and a highly significant positive correlated with the numbers of healthy piglets (*p* < 0.01). Fecal non-targeted metabolomics revealed that differential metabolites were significantly enriched in bile secretion and prolactin signaling pathways, with a series of bile acids, including hyodeoxycholic acid (HDCA), chenodeoxycholic acid (CDCA), glycochenodeoxycholic acid (GCDCA), cholic acid (CA), lithocholic acid (LCA), ursodeoxycholic acid (UDCA) and γ-muricholic acid (γ-MCA), were significantly increased in the DF group. And the abundance of *NK4A214* was positively correlated with GCDCA (*p <* 0.05) and progesterone (*p <* 0.01).

**Conclusion:**

The abundance of *Oscillospiraceae*, especially *NK4A214_group* of DF sows during gestation, may improve the numbers of total born and healthy piglets, with GCDCA likely playing a significant role in this process.

## 1 Introduction

It is important to maximize the reproductive performance of sows in the swine industry. Owing to the genetic selection and improvements in health, management and nutrition, sow productivity has led to remarkably high levels (Tokach et al., 2019). However, the metabolic disorders during perinatal period such as insulin resistance and systemic low-grade inflammation, which may be related to gut microbes (Cheng et al., 2018), adversely impact reproductive performance of sow (Cheng et al., 2020). Accumulating evidence suggests that these metabolic disturbances are closely linked to changes in the gut microbiota, which plays a central role in regulating host energy metabolism, immune homeostasis, and inflammatory responses (Cheng et al., 2018; Giugliano et al., 2025). Therefore, it is crucial to develop effective nutritional strategies that can modulate gut microbiota in order to enhance sow reproductive performance.

In recent decades, increasing studies have shown that gut microbiome plays key roles in reproductive performance of sows (Cheng et al., 2018; Chen et al., 2023; Liu et al., 2023; Chen et al., 2025). For example, bile acid-metabolizing bacteria have attracted attention, as bile acids act as both digestive molecules and signaling metabolites that regulate glucose metabolism, inflammation and reproductive hormone homeostasis (Wahlström et al., 2016; Fuchs and Trauner, 2022). Gut commensals such as *Lactobacillus reuteri* and Prevotella spp. have been implicated in regulating reproductive hormone metabolism, including estrogen and progesterone pathways, which may facilitate estrus return and reproductive success (Liu et al., 2023). Furthermore, genera associated with dysbiosis, such as Treponema, have been reported to correlate with adverse reproductive outcomes in sows (Sanglard et al., 2020; Chen et al., 2023). Together, these findings emphasize the importance of both microbial composition and microbial-derived metabolites in reproductive success.

Dietary fiber is a well-recognized modulator of gut microbiota composition and function and has been widely applied in sow nutrition to alleviate oxidative stress, reduce inflammatory responses, and improve reproductive performance (Jiang et al., 2019; Xu et al., 2020; Zhuo et al., 2020; Shang et al., 2021; Li et al., 2023). For instance, sows fed the gestation diets supplementation with inulin had lower endotoxin concentrations, and improved sow and litter performance (Li et al., 2021). Besides, the microbial composition and diversity in inulin group were changed significantly (Zhou et al., 2017). However, whether fibers can enhance the stability and resilience of the gut microbiota across critical reproductive stages, rather than merely shifting microbial abundance, has not been systematically investigated.

In the current study, gestation diets of sows were supplemented with fiber to investigate the effects on reproductive performance and microbial composition. This study will reveal a novel perspective on improving reproductive performance in sows by focusing on the stability of the gut microbiota, highlighting the importance of maintaining a balanced microbial ecosystem for optimal reproductive outcomes.

## 2 Materials and methods

### 2.1 Animals and experimental design

**Figure 1:**
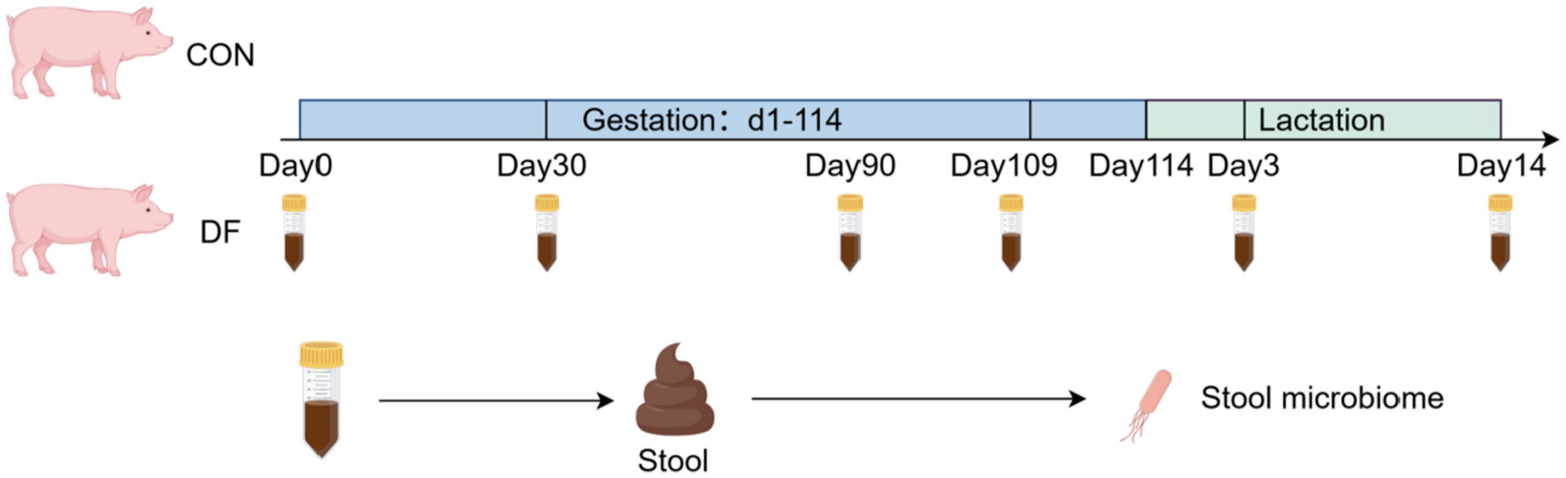
Experimental design.

The animal study was reviewed and approved by the Animal Care and Use Committee of Huazhong Agricultural University. The overall design of the experiment is illustrated in Figure 1. This experiment was conducted at the Wuli Town pig farm, Xin Yang of Tecon Biology Co. A total of 1,000 healthy sows of comparable body weight (DanBred Landrace × DanBred Yorkshire, parities 1–2) were selected and randomly assigned to two dietary treatment groups after artificial insemination. Sows in the control (CON) group received a diet primarily composed of beet pulp and barley as fiber sources, while those in the experimental group were provided a diet supplemented with 1% functional fiber [DF, consisted of 85.7% resistant starch (Hangzhou, China) and 14.3% guar gum (Yunzhou, China)]. The functional fiber was provided by Wuhan Fanbo Biotechnology Co., Ltd. (Wuhan, China). This was achieved by partially replacing the energy-yielding ingredients in the control diet while ensuring that the net energy levels in the CON and DF diets was comparable. All sows were housed in individual gestation stalls (2.2 m length × 0.65 m width × 0.6 m height). Sow culling data were recorded during gestation (Supplementary Table S1).

All experimental diets were formulated to meet or exceed the nutritional requirements for gestating and lactating sows as outlined in National Research Council (NRC) (2012) (Supplementary Table S2). The gestation diet was provided at 2.6 kg/d from days 0 to 30, 2.2 kg/d from days 31 to 90, and 2.8 kg/d from days 91 to 109. From day 110 until the day before parturition, sows received 2.0 kg/d. Post-farrowing, all sows received the same lactation diet throughout the lactation period, and sows were fed 1.0 kg/d on the first day postpartum, with the amount increased by 1.0 kg/d over the next 7 days. Thereafter, sows had ad libitum access to feed until weaning.

### 2.2 Measurements of reproductive performance

Sow performance and reproductive parameters were recorded during gestation and lactation. These included total piglets born, live-born piglets, birth weight, stillbirths, mummified fetuses, number of piglets weaned, weaning weight, and the interval to return to estrus. Reproductive performance data were collected, encompassing the number of total piglets born, piglets born alive, healthy piglets (birth weight ≥ 0.8 kg), piglets with intrauterine growth retardation (IUGR) (birth weight < 0.8 kg), piglets born dead (stillborn, mummified, crushed, or abnormal), litter birth weight, and individual piglet birth weight.

### 2.3 Collection of fecal samples

Fecal specimens were systematically collected from 20 randomly selected sows per group at critical developmental stages. In detail, fresh fecal samples (~5 g/sow) were collected at gestation days 0 (G0 d), 30 (G30 d), 90 (G90 d), and 109 (G109 d), as well as lactation days 3 (L3 d) and 14 (L14 d). All samples were immediately frozen in liquid nitrogen and stored at −80 °C until further analysis.

### 2.4 Microbial DNA extraction

#### 2.4.1 16S rRNA gene sequencing and bioinformatics analysis

Microbial DNA was extracted from frozen feces using a QIAamp DNA Stool Mini Kit (Qiagen, Germany) following the manufacturers protocols. The quality and concentration of DNA were determined by 1.0% agarose gel electrophoresis and a NanoDrop® ND-2000 spectrophotometer (Thermo Scientific Inc., United States) and kept at −80 °C prior to further use.

The V3–V4 hypervariable region of the bacterial 16S rRNA gene was amplified using primers 338F (5-ACT CCT ACG GGA GGC AGC AG-3) and 806R (5-GGA CTACHV GGG TWT CTA AT-3) by an ABI GeneAmp® 9700 PCR thermocycler (ABI, CA, United States). Sequencing and data analysis were subsequently performed on an Illumina MiSeq PE300 platform platform (Illumina, San Diego) according to the standard protocols by Majorbio Bio-Pharm Technology Co. Ltd. (Shanghai, China). After demultiplexing, the resulting sequences were quality filtered with fastp (0.19.6) (Chen et al., 2018) and merged with FLASH (v1.2.11) (Magoc and Salzberg, 2011). Then the high-quality sequences were de-noised using DADA2 (Callahan et al., 2016) plugin in the Qiime2 (Bolyen et al., 2019) (version 2020.2) pipeline with recommended parameters. Finally, a feature table of amplicon sequence variants (ASVs) was obtained for downstream analyses. To minimize the effects of sequencing depth on alpha and beta diversity measure, the number of sequence from each sample was rarefied to 23,637, which still yielded an average Good’s coverage of 99.73%. Taxonomic assignment of ASVs was performed using the Naive bayes consensus taxonomy classifier implemented in Qiime2 and the SILVA 16S rRNA database (v138).

#### 2.4.2 Untargeted metabolome of feces samples

The fecal samples were thawed on ice. A 60 mg fecal sample was combined with 1 mL of cold 90% methanol. The lysate was homogenized using an MP homogenizer (24 × 2, 6.0 m/s, 60 s, twice) and sonicated at low temperature (30 min per cycle, twice). The homogenate was centrifuged at 14,000 g for 20 min at 4 °C. The supernatant was dried in a vacuum centrifuge. For LC–MS analysis, the dried samples were reconstituted in 100 μL of acetonitrile/water (1:1, v/v) solvent. To monitor the stability and repeatability of instrument analysis, quality control (QC) samples were prepared by pooling 10 μL of each sample and analyzed together with the other samples. The QC samples were inserted regularly and analyzed every 5 samples.

Analysis was performed using an UHPLC (Vanquish UHPLC, Thermo) coupled to an Orbitrap (Q Exactive HF-X/Q Exactive HF) in Shanghai Applied Protein Technology Co., Ltd. For HILIC separation, samples were analyzed using a 2.1 mm × 100 mm ACQUIY UPLC BEH Amide 1.7 μm column (Waters, Ireland). In both ESI positive and negative modes, the mobile phase contained A = 25 mM ammonium acetate and 25 mM ammonium hydroxide in water and B = acetonitrile. The gradient was 98% B for 1.5 min and was linearly reduced to 2% in 10.5 min, and then kept for 2 min, and then increased to 98% in 0.1 min, with a 3 min re-equilibration period employed.

The ESI source conditions were set as follows: Ion Source Gas1 (Gas1) as 60, Ion Source Gas2 (Gas2) as 60, curtain gas (CUR) as 30, source temperature: 600 °C, IonSpray Voltage Floating (ISVF) ± 5,500 V. In MS only acquisition, the instrument was set to acquire over the m/z range 80–1,200 Da, the resolution was set at 60,000 and the accumulation time was set at 100 ms. In auto MS/MS acquisition, the instrument was set to acquire over the m/z range 70–1,200 Da, the resolution was set at 30,000 and the accumulation time was set at 50 ms, excluding time within 4 s.

The raw MS data were converted to MzXML files using ProteoWizard MSConvert before importing into freely available XCMS software. For peak picking, the following parameters were used: centWave m/z = 10 ppm, peakwidth = c (10, 60), prefilter = c (10, 100). For peak grouping, bw = 5, mzwid = 0.025, minfrac = 0.5 were used. CAMERA (Collection of Algorithms of MEtabolite pRofile Annotation) was sued for annotation of isotopes and adducts. In the extracted ion features, only the variables having more than 50% of the nonzero measurement values in at least one group were kept. Compound identification of metabolites was performed by comparing of accuracy m/z value (<10 ppm), and MS/MS spectra with an in-house database established with available authentic standards.

### 2.5 Co-occurrence network construction and topological analysis

Microbial co-occurrence networks were constructed at the genus level using SparCC with 100 bootstrap iterations and a significance threshold of *p* < 0.05, as implemented in the SpiecEasi R package (v1.1.2) (Friedman and Alm, 2012). Only correlations with |r| > 0.5 were retained to build the final networks (Chen et al., 2023). Network topological properties were calculated using Cytoscape (v.3.10.2) (Shannon et al., 2003). Network visualizations were generated in Gephi (v.0.9.2) (Bastian et al., 2009). Natural connectivity analysis was performed following the method described by Pan et al. (2021), and the visualization of natural connectivity dynamics was generated using the ggplot2 package (version 3.5.1) in R. Natural connectivity (Wu et al., 2010) was introduced to describe network stability differences, the estimation of natural connectivity was based on the following algorithm:


$$\mathrm{ave}.(k)=\ln\;((1/N)\sum_{i=1}N\;e^{\mathrm{ki}})$$

where ave.(k) represents the natural connectivity, N is the number of nodes in the network, and kᵢ denotes the eigenvalue of the adjacency matrix.

### 2.6 Statistical analysis

The performance and reproductive parameters of sows were analyzed separately for the full dataset (Table 1) and the microbiome subset (Table 2). For the Table 1 (CON: *n* = 405; DF: *n* = 411), a one-way analysis of variance (ANOVA) was performed using the GLM procedure in SAS (version 9.4; SAS Institute Inc., Cary, NC, United States), with dietary treatment (CON vs. DF) as the fixed effect and individual sows as the experimental units. Parity was not included as a covariate due to the large sample size and the relatively balanced distribution between groups (CON: 1.59 ± 0.68; DF: 1.79 ± 0.66).

**Table 1:** Effects of dietary supplementation with DF on litter performance of sows.

Item	CON	DF	*P*-value
No. of sows	405	411	
Parity	1.59 ± 0.68	1.79 ± 0.66	
Total born, *n*	16.55 ± 4.55	17.59 ± 4.23	*P <* 0.01
Born alive, *n*	15.16 ± 4.02	15.58 ± 3.66	0.14
Healthy piglets, *n*	14.53 ± 3.8	15.22 ± 3.53	*P <* 0.01
IUGR, *n*	0.62 ± 1.11	0.36 ± 0.83	*P <* 0.01
White stillbirth, *n*	1.04 ± 1.53	1.32 ± 1.76	*P <* 0.05
Black stillbirth, *n*	0.16 ± 0.52	0.44 ± 0.96	*P <* 0.01
Number of mummified fetus	0.17 ± 0.77	0.23 ± 0.65	0.15
Number of abnormal piglet	0.02 ± 0.15	0.01 ± 0.12	0.38
Litter birth weight, kg	19.26 ± 4.91	20.13 ± 4.7	*P <* 0.01
Individual average weight, kg	1.30 ± 0.21	1.31 ± 0.2	0.31

CON, control diet; DF, functional fiber diet; Data were analyzed by one-way ANOVA with diet as the fixed effect. Parity did not differ significantly between groups and was therefore not included as a covariate.

**Table 2:** Effects of dietary supplementation with DF on reproductive performance of the selected sows (*n* = 16 per group).

Item	CON	DF	*P*-value
No. of sows	16	16	
Total number of born	15.81 ± 2.70	20.25 ± 2.11	0.001
Number of born alive	15.00 ± 2.32	17.63 ± 2.23	0.01
Number of born healthy	14.50 ± 2.18	17.31 ± 2.08	0.01

For exploratory microbiome and metabolome analyses, a subset of 32 sows (*n* = 16 per group) was selected based on extreme reproductive performance. Following an initial random sampling of 20 sows per group, the 16 sows with the lowest total number of piglets born (CON group) and the 16 sows with the highest total number of piglets born (DF group) were chosen to amplify potential differences associated with treatment. Comparisons between CON and DF groups in this subset were performed using an unpaired two-tailed Student’s *t*-test (GraphPad Prism, version 8.3.0). It should be noted that selecting sows based on extreme reproductive performance may introduce selection bias. This exploratory subset analysis aimed to characterize diet-associated microbial differences, and the results should be interpreted as supportive, hypothesis-generating evidence rather than as definitive inferences at the population level.

Alpha and beta diversity of microbial data were calculated using the vegan package (version 2.6–8) in R software (version 4.1.3). PCoA was performed using Bray-Curtis distance metrics. PERMANOVA was used to evaluate factors shaping microbiota by using the Adonis function of the vegan package (999 permutations). In addition, the significantly different genera were selected by the linear discriminant analysis effect size (LEfSe) method (Segata et al., 2011), performed via Wekemo Bioincloud^1^ (Gao et al., 2024).

Orthogonal partial least squares discriminant analysis (OPLS-DA) was also introduced as a supervised model to reveal the metabolome variation between groups, using the “plsda” function in R package “ropls” (version 1.26.4). The analysis was conducted on untargeted metabolomics. The differential metabolites with false discovery rate (*FDR*) < 0.05 and the absolute value of |log2 (fold change)| > 1 were visualized by a volcano plot. The results were visualized using the ggplot2 (version 3.5.1) package for R. Statistical significance was determined when *p* value was < 0.05.

## 3 Results

### 3.1 DF treatment improves reproductive performance of sows

As shown in Table 1, compared with the CON group, the numbers of total born and healthy piglets were significantly improved (*p* < 0.01) in the DF group, accompanied by an increase in litter birth weight (*p* < 0.05). The number of IUGR piglets was significantly reduced (*p* < 0.05) in DF group. However, the numbers of white and black stillbirths were also higher in the DF group (*p* < 0.05). No significant differences were observed between the CON and DF groups in the numbers of piglets born alive, mummified fetuses, abnormal piglets, or average birth weight per piglet (*p* > 0.05).

### 3.2 DF treatment enhances the gut microbial stability of sows during gestation

**Figure 2:**
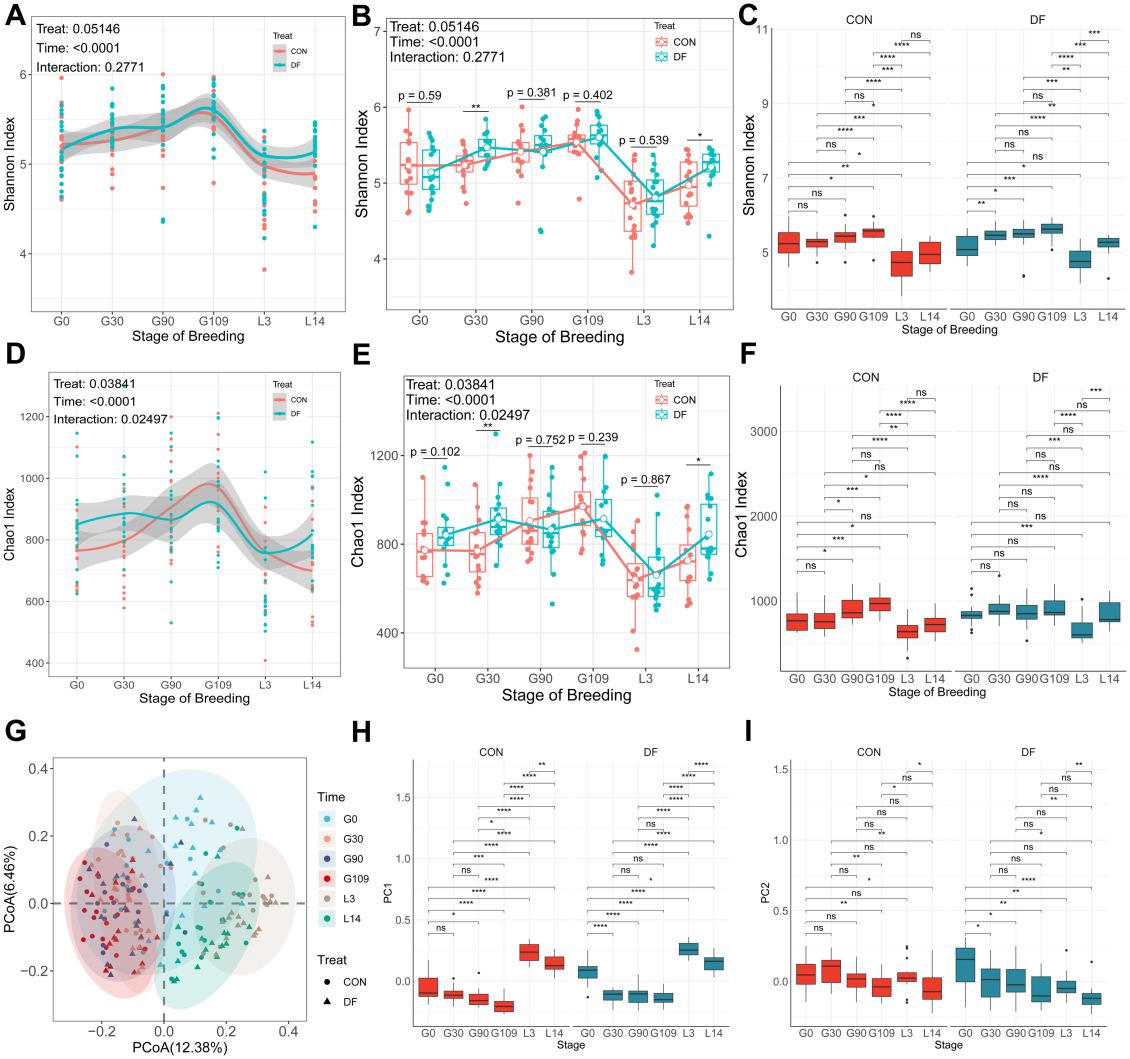
DF treatment enhances stability of gut microbiota during gestation and accelerates recovery of microbiota during lactation in sows. **(A–C)** Shannon index. **(D–F)** Chao1 index. **(G)** PCoA of the gut microbiota in sows based on Bray-Curtis distances. **(H,I)** PC1 and PC2. In panels **(A–F,H,I)** red represents the CON group, and blue represents the DF group. Results are expressed as median and quartile. *p* < 0.05 indicates statistical significance (*p < 0.05, **p < 0.01, ***p < 0.001, and ****p < 0.0001).

In order to study the composition of gut microbiota in sows with different reproductive performance, fecal samples from 32 sows with significantly different reproductive outcomes (Table 2) were subjected to 16S rRNA gene sequencing. Global changes in the gut microbial community were observed by assessing diversity. Microbial diversity (Shannon index) and richness (the Chao1 estimator, Chao1) initially increased, subsequently declined, and later exhibited a recovery (Figures 2A,D). To assess the effects of dietary treatment and time on microbial α-diversity, linear mixed-effects models were employed using the nlme package (version 3.1–162) in R. These models were chosen because they appropriately account for the longitudinal and repeated-measures design of the study. The analysis revealed microbial α-diversity exhibited significant changes over time (*p* < 0.0001). Dietary treatment also influenced the microbiota, showing a trend toward significance for Shannon diversity (*p* = 0.051; Figures 2A,B) and a significant effect on richness as estimated by Chao1 (*p* = 0.038; Figures 2D,E). The alpha diversity of DF microbiota was higher than CON on G30 d and L14 d (*p* < 0.05, Figures 2B,E). Incorporating the changes in diversity over time, the diversity in the DF group significantly increased at G30 d and remained stable, while the CON group gradually increased with the progression of pregnancy (*p* < 0.05, Figure 2C). Regarding the Chao1 index, the DF group exhibited an increase in the median value from G0 d to G30 d, although the change was not statistically significant, while the CON group remained relatively stable. Consequently, the results showed a significant difference between the two treatments at G30 d, with no significant difference observed at G109 d. These findings suggest that sows in the DF group rapidly achieved a stable gut microbial community, indicating that DF supplementation enhanced the stability of the gut microbiota. On the other hand, microbial diversity significantly decreased in both treatment groups after farrowing in sows (*p* < 0.05, Figures 2C,F); however, the diversity in the DF group significantly increased from L3 d to L14 d and showed no significant difference compared to G0 d, whereas the CON group, despite a significant increase at L14 d compared to L3 d, remained significantly lower than G0 d (Figure 2C). These results indicate that DF treatment facilitated, to a certain degree, the recovery of the gut microbiota during lactation.

For beta diversity, Principal coordinate analysis (PCoA) based on Bray–Curtis dissimilarity revealed that the structure of the gut microbiota was distinctly differentiated by different stages of gestation (Figure 2G), particularly with the gestation and lactation periods clearly clustering into two separate groups (Supplementary Figure S1A). And PCoA exhibited significant segregation except on L3 d (PERMANOVA, *p* < 0.05, Supplementary Figure S1B). PC1 and PC2 analyses revealed similar microbial trajectory patterns between sows in the CON and DF group (Figures 2H,I). Consistent with the changes in α-diversity, at G30 d, both PC1 and PC2 values in the DF group significantly decreased and remained stable at subsequent gestation time points, whereas the CON group exhibited a continuous decline throughout the gestation period (Figures 2H,I).

### 3.3 Temporal dynamics of the microbial community

**Figure 3:**
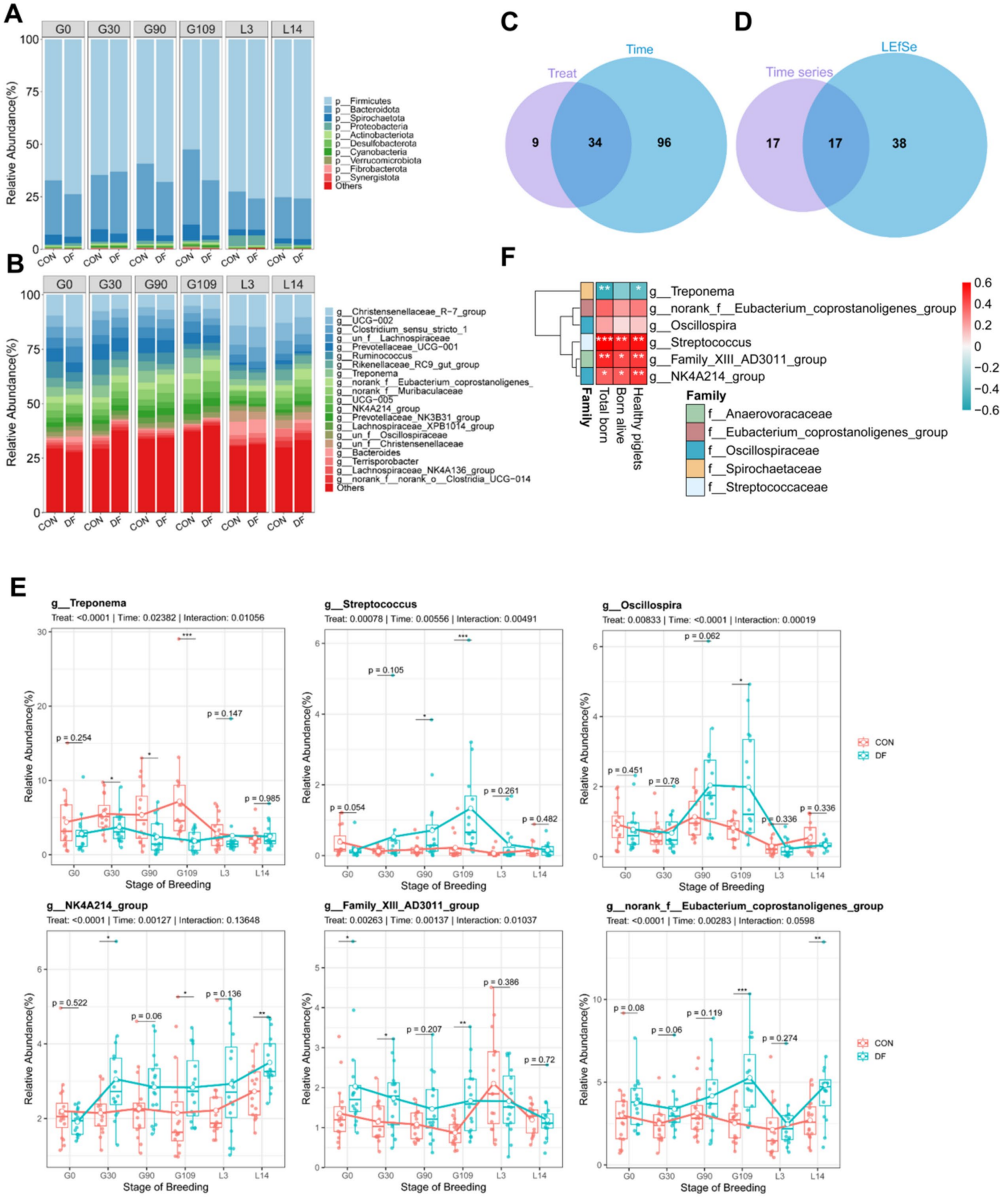
Specific bacterial taxa selectively promoted by functional fiber may be associated with the improvement of reproductive performance in sows. **(A)** Phylum level. **(B)** Genus level. Venn diagrams illustrating **(C)** the overlap between genera with significant differences in treatment and stage identified by the linear mixed-effects model (LMM), and **(D)** the intersection of these taxa with those identified by LEfSe analysis. **(E)** Differential genera identified by both LMM and LEfSe analyses with relative abundance > 0.1%. **(F)** Relationships between the overlapping taxa identified in panel E and reproductive performance. Spearman’s correlation coefficients were calculated to assess the associations between bacterial genera and reproductive performance parameters at G109d. Color intensity represents the correlation coefficient (*ρ*). Results are expressed as median and quartile. *p* < 0.05 indicates statistical significance (*p < 0.05, **p < 0.01, ***p < 0.001, and ****p < 0.0001).

The relative abundances of the top 10 phyla (Figure 3A) and top 20 genera (Figure 3B) were calculated as the overall mean across all samples, providing a comprehensive overview of fecal microbial composition. The gut microbial community was dominated by *Firmicutes* (68.33%) and *Bacteroidota* (23.65%), followed by *Spirochaetota* (3.78%), *Proteobacteria* (1.94%) and *Actinobacteriota* (0.6%) (Figure 3A). Likewise, *Christensenellaceae_R-7_group* (9.58%), *UCG-002* (5.72%), *Clostridium_sensu_stricto_1* (4.86%), *un_f__Lachnospiraceae* (4.51%), *Prevotellaceae_UCG-001* (4.08%), *Ruminococcus* (3.74%), *Rikenellaceae_RC9_gut_group* (3.74%), *Treponema* (3.61%), *norank_f__Eubacterium_coprostanoligenes_group* (3.32%) and *norank_f__Muribaculaceae* (3.31%) were the 10 most abundant genera (Figure 3B). In order to evaluate the impact of treatment on the composition of the gut microbiota at each gestation stage, we compared the relative abundance of the Firmicutes and Bacteroidetes phyla, as well as the Firmicutes-to-Bacteroidetes ratio, in the CON and DF groups. Significant alterations were observed only on gestation day 109 (Supplementary Figure S2). Specifically, compared with the CON group, the abundance of *Firmicutes* was markedly increased in the DF group (*p* < 0.001, Supplementary Figure S2A), whereas the abundance of *Bacteroidetes* was significantly decreased (*p* < 0.05, Supplementary Figure S2B). Consequently, the *Firmicutes*-to-*Bacteroidetes* ratio was significantly elevated in the DF group (*p* < 0.01, Supplementary Figure S2C), indicating that the gut microbial composition was markedly altered.

To evaluate the temporal dynamics of the microbial community across reproductive stages and dietary treatments, we applied a linear mixed-effects model at the genus level (Supplementary Table S3). A total of 43 genera exhibited significant differences in response to dietary treatment, 130 genera showed significant temporal variation across reproductive stages, and 23 genera were significantly influenced by the treatment × stage interaction. Of these, 34 genera were simultaneously affected by both treatment and stage (Figure 3C; Table 3), and were considered key genera.

**Table 3:** Genera with significant treatment and time effects identified by linear mixed-effects models.

Genera	Treatment	Time	Interaction
*g__Akkermansia*	0.0298	0.0012	0.5237
*g__Anaerovorax*	0.0481	0.0000	0.4944
*g__Bacillus*	0.0407	0.0117	0.0637
*g__Cellulosilyticum*	0.0001	0.0000	0.0000
*g__Erysipelotrichaceae_UCG-003*	0.0324	0.0122	0.7026
*g__Eubacterium_hallii_group*	0.0264	0.0000	0.0872
*g__Eubacterium_nodatum_group*	0.0000	0.0026	0.2233
*g__Eubacterium_siraeum_group*	0.0206	0.0000	0.8968
*g__Family_XIII_AD3011_group*	0.0026	0.0014	0.0104
*g__Family_XIII_UCG-001*	0.0063	0.0000	0.0161
*g__Frisingicoccus*	0.0004	0.0184	0.3211
*g__Lachnospiraceae_FCS020_group*	0.0001	0.0001	0.1086
*g__Lachnospiraceae_NK4B4_group*	0.0019	0.0002	0.0020
*g__Marvinbryantia*	0.0009	0.0000	0.0439
*g__Moryella*	0.0005	0.0000	0.0050
*g__NK4A214_group*	0.0001	0.0013	0.1365
*g__norank_f__Eubacterium_coprostanoligenes_group*	0.0000	0.0028	0.0598
*g__Olsenella*	0.0436	0.0070	0.2750
*g__Oscillospira*	0.0083	0.0000	0.0002
*g__Peptococcus*	0.0031	0.0000	0.4936
*g__Prevotellaceae_UCG-001*	0.0497	0.0000	0.9902
*g__Roseburia*	0.0177	0.0021	0.4968
*g__Ruminococcus_gauvreauii_group*	0.0002	0.0005	0.0441
*g__Streptococcus*	0.0008	0.0056	0.0049
*g__Subdoligranulum*	0.0279	0.0077	0.0801
*g__Treponema*	0.0000	0.0238	0.0106
*g__UCG-005*	0.0000	0.0006	0.0279
*g__un_f__Eggerthellaceae*	0.0000	0.0001	0.4219
*g__un_f__Erysipelotrichaceae*	0.0051	0.0003	0.0038
*g__un_f__Oscillospiraceae*	0.0169	0.0185	0.2193
*g__un_f__Ruminococcaceae*	0.0001	0.0006	0.4380
*g__un_o__Bacteroidales*	0.0025	0.0000	0.1607
*g__un_o__Coriobacteriales*	0.0000	0.0000	0.0061
*g__XBB1006*	0.0004	0.0002	0.2397

Microbial differences between dietary treatments during gestation were identified using linear discriminant analysis (LDA) coupled with effect size estimation (LEfSe). As shown in Supplementary Table S4, using an LDA score > 2 as the threshold, 28 genera were significantly different at G0 d, 23 genera at G30 d, 24 genera at G90 d, and 55 genera at G109 d. These results indicated that G109d had the largest number of differentially abundant genera, reflecting the greatest compositional differences between the CON and DF groups. At G109d specifically, these 55 genera comprised 18 taxa significantly enriched in the CON group and 37 taxa enriched in the DF group. Therefore, the differential genera identified by LEfSe at G109 d were further integrated with the key genera revealed by the linear mixed-effects model (Figure 3D). In total, 17 genera were identified as differential taxa by both approaches, and their relative abundances were shown in Figure 3E and Supplementary Figure S3. The genera with relative abundance > 0.1% were shown in Figure 3E. During early gestation (G0-G30d), distinct microbial differences between the CON and DF groups were already evident. Treponema showed a significant increase in the CON group starting from G30d (*p <* 0.05). *Streptococcus* showed a tendency toward higher abundance at G0d (*p <* 0.1) in DF group. Compared with the CON group, *Family_VIII_AD3011_group* exhibited significantly higher abundance at G0d (*p <* 0.05) and G30d (*p <* 0.05), while *norank_f_Eubacterium_coprostanoligenes_group* showed a tendency toward higher abundance at G0d (*p <* 0.1) and a significant increase at G30d (*p <* 0.05). Moreover, *NK4A214_group* was significantly increased in the DF group at G30d (*p <* 0.05), indicating an early microbial response to DF diet.

As gestation progressed into late pregnancy (G90-G109d) and lactation (L3-L14d), these microbial differences became more pronounced. *Treponema* remained significantly enriched in the CON group during late gestation (*p <* 0.05). In the DF group, *Streptococcus* was significantly enriched during late pregnancy (*p* < 0.05). And *Oscillospira* showed a tendency toward higher abundance at G90d (*p <* 0.1) and a significant increase at G109d (*p <* 0.05) compared to the CON group. *Family_VIII_AD3011_group* and *norank_f_Eubacterium_coprostanoligenes_group* continued to show higher abundances in the DF group and were both significantly enriched at G109d (*p <* 0.01 and *p <* 0.001, respectively), with *norank_f_Eubacterium_coprostanoligenes_group* also exhibiting significantly higher abundance at L14d (*p <* 0.01). Furthermore, *NK4A214_group* maintained a relatively high abundance throughout the entire reproductive period, showing a trend toward higher abundance at G90d (*p* < 0.1), a significant increase at G109d (*p* < 0.05), and a further increase at L14d (*p* < 0.01) compared with the CON group. Overall, the temporal dynamics of these genera suggest that *Treponema* and *NK4A214_group* may serve as key differential genera between the CON and DF groups, reflecting distinct microbial trajectories under different dietary treatments.

The G109d time point was selected for association analysis with reproductive performance because previous study has indicated that the gut microbiome undergoes the most profound remodeling during late gestation, which supports metabolic adaptations beneficial for fetal development and litter size (Koren et al., 2012; Chen et al., 2023). Spearman correlation analysis was performed to investigate the relationship between the bacterial genera abundance at G109 d and reproduction performance of sows (Figure 3F). Among the genera with high abundance, the relative abundance of *Treponema* was negatively correlated with the numbers of total born (*p* < 0.01) and healthy piglets (*p* < 0.05, Figure 3F). The relative abundance of *Streptococcus* showed a highly significant positive correlated with the numbers of total born, born alive and healthy piglets (*p* < 0.01). The relative abundance of *Family_VIII_AD3011_group* was highly positively correlated with the numbers of total born and healthy piglets (*p* < 0.01), and positively correlated with the numbers of born alive (*p* < 0.05, Figure 3F). In addition, *NK4A214_group* showed a positive correlated with the numbers of total born and born alive (*p* < 0.05), and a highly significant positive correlated with the numbers of healthy piglets (*p* < 0.01, Figure 3F).

Moreover, we applied the same analytical strategy at the family level (Supplementary Figure S4; Supplementary Tables S5, S6). A total of 13 families were significantly influenced by both treatment and stage (Supplementary Figure S4A; Supplementary Table S5). And the Venn diagram showed 7 families were identified as differential taxa by both the linear mixed-effects model and LEfSe analysis (Supplementary Figure S4B), and their relative abundances were shown in Supplementary Figure S4C. The correlation heatmap indicated that *Spirochaetaceae* was significantly negatively correlated with the numbers of total born (*p* < 0.01) and healthy piglets (*p* < 0.05, Supplementary Figure S4D), which was enriched in the CON group (Supplementary Figure S4C). In addition, among the 6 families that were enriched in the DF group (Supplementary Figure S4C), *Oscillospiraceae*, the highest abundance with a median relative abundance of 14.74%, was markedly positively correlated with the numbers of total born and healthy piglets (*p* < 0.05, Supplementary Figure S4D). Taken together, these findings suggest that *Spirochaetaceae*, especially *Treponema*, are negatively associated with reproductive performance, whereas *Oscillospiraceae* and its member *NK4A214_group* enriched in DF group may play a positive role in improving reproductive performance of sows.

### 3.4 The stability and major topological properties of the microbial co-occurrence networks

**Figure 4:**
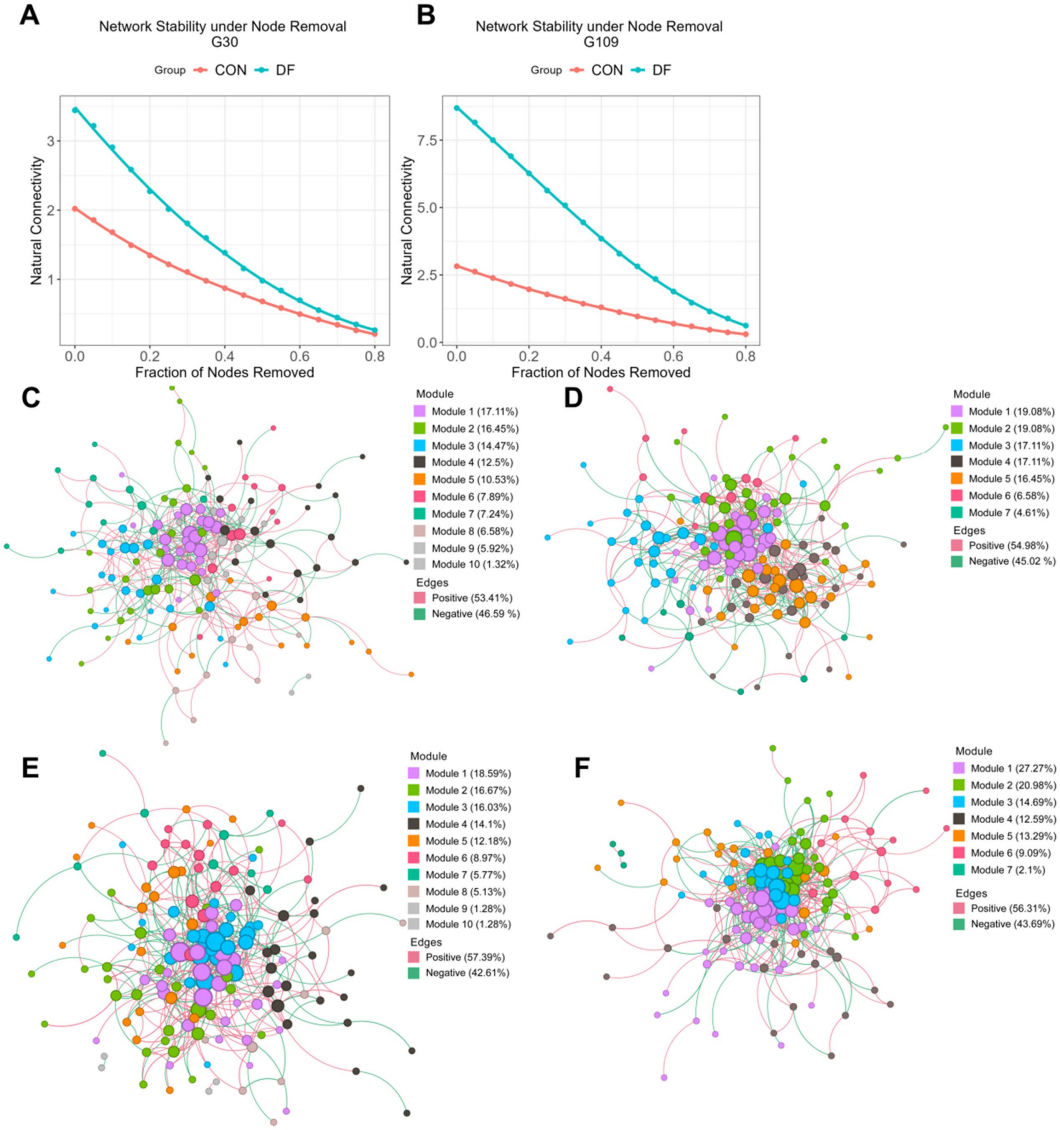
Microbial co-occurrence network and network stability at G30 and G109. Network stability at **(A)** G30 d and **(B)** G109 d. Co-occurrence networks for the CON group at **(C)** G30 d and **(E)** G109 d, corresponding networks for the DF group at **(D)** G30 d and **(F)** G109 d. Nodes in the network represent taxon (genus level).

**Figure 5:**
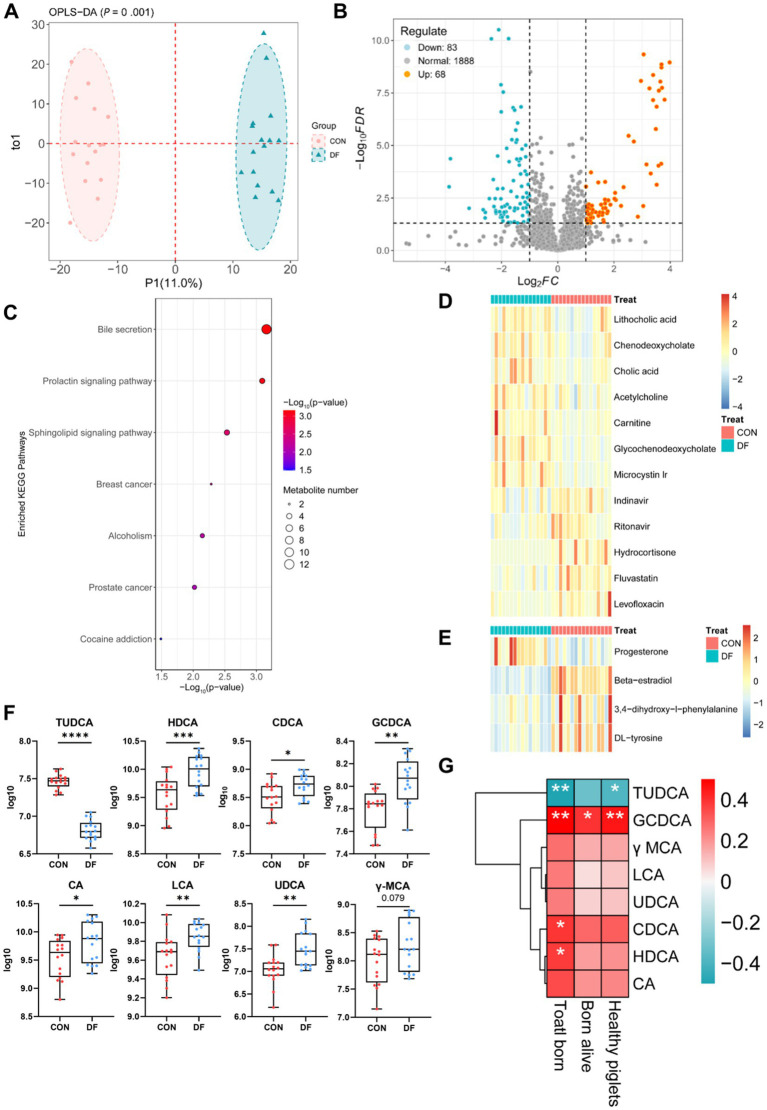
Effects of DF treatment on fecal metabolites in sows. **(A)** Orthogonal partial least squares discriminant analysis (OPLS-DA) of metabolites in sows with CON and DF. p values of the model are calculated by 1,000 permutations. **(B)** Volcano plot of the metabolites. **(C)** KEGG pathway enrichment analysis of the differential metabolites. **(D)** Heatmap of metabolites in the bile secretion pathway. **(E)** Heatmap of metabolites in the prolactin signaling pathway. **(F)** Comparison of TUDCA, HDCA, CDCA, GCDCA, CA, LCA, UDCA, and y-MCA levels between the two groups. **(G)** Spearman’s correlation coefficients were calculated to assess the associations between bile acids and reproductive performance at G109d. Color intensity represents the correlation coefficient (*ρ*). *p* < 0.05 indicates statistical significance (*p < 0.05, **p < 0.01, ***p < 0.001, and ****p < 0.0001).

Furthermore, based on the patterns of diversity dynamics (Figure 2) and differential taxa analysis (Figure 3), we consider G30 d and G109 d to be the most important stages during gestation. Therefore, Co-occurrence networks were constructed using SparCC to evaluate the network properties and stability at these two time points (Figure 4). The network stability at G30 d and G109 d was shown in Figures 5A,B, respectively, where the DF group consistently exhibited higher stability than the CON group, with an obvious increase from G30 d to G109 d. Co-occurrence networks constructed for the CON and DF groups at G30 d and G109 d were illustrated in Figures 4C–F, and the topological properties of the networks were presented in Table 4. We found that the number of nodes among the four networks was comparable, but the numbers of edges and average number of neighbors were obviously different. To be more specific, compared with CON group, both metrics were higher in DF group, with more edges (521 vs. 425 at G30; 737 vs. 542 at G109) and a greater average number of neighbors (6.83 vs. 5.65 at G30; 10.5 vs. 7.13 at G109), which signify the connectivity of network in the DF group was stronger than CON group. Moreover, the clustering coefficient, network density, and network centralization exhibited similar patterns, showing comparable values between the groups at G30 d, whereas all three metrics were higher in the DF group than in the CON group at G109 d (clustering coefficient: 0.358 vs. 0.273; network density: 0.076 vs. 0.047; network centralization: 0.186 vs. 0.106). Collectively, these results indicate that DF treatment enhanced microbial network connectivity and clustering, leading to tighter internal organization, which may partly underlie the observed differences in network stability.

**Table 4:** Topological properties of co-occurrence networks of microbial community.

Parameters	CON_G30	DF_G30	CON_G109	DF_G109
Number of nodes	153	147	148	144
Number of edges	434	492	517	779
Avg. number of neighbors	5.735	6.806	6.986	10.958
Characteristic path length	3.439	3.271	3.21	2.843
Clustering coefficient	0.243	0.252	0.247	0.38
Network density	0.038	0.048	0.048	0.078
Network heterogeneity	0.782	0.816	0.668	0.925
Network centralization	0.123	0.122	0.076	0.216

To further characterize the core network structure, the importance of each node was evaluated using MCC scores (Table 5; Supplementary Table S7). In the CON group, *g__NK4A214_group* exhibited very high MCC scores at both G30 d and G109 d, indicating a consistently central role in the microbial network. And *g__Treponema* also showed relatively high MCC rankings in the CON networks, particularly at G109 d. By contrast, in the DF group, both the *g__NK4A214_group* and the *g__Treponema* displayed substantially lower MCC rankings at G30 d (ranked 39/147 and 41/147, respectively), suggesting reduced centrality during the early stages of gestation. By G109 d, *g__Treponema* showed an increased MCC ranking (15/144), whereas *g__NK4A214_group* remained low in centrality, indicating treatment-specific differences in core network organization.

**Table 5:** Keystone taxa ranking based on MCC scores in four microbial co-occurrence networks.

Network	Metric	*g__NK4A214_group*	*g__Treponema*
CON_G30	MCC scores	85	10
	Rank	4/153	25/153
CON_G109	MCC scores	19	17
	Rank	6/148	10/148
DF_G30	MCC scores	6	6
	Rank	39/147	41/147
DF_G109	MCC scores	4	196
	Rank	82/144	15/144

### 3.5 Fecal metabolite profiles of sows

To characterize the metabolome signatures of sows on G109 d, the fecal metabolic profiles were analyzed by non-targeted LC–MS/MS metabolomics. Overall, stool metabolite profiles were found to significantly differ between the CON and DF group by OPLS-DA (*p* = 0.001; Figure 5A). Compared with CON group sows, DF group sows displayed increased concentrations of 68 metabolites and decreased concentrations of 83 metabolites (Figure 5B). KEGG analysis revealed that most of the differential metabolites in DF group compared with CON group were enriched in bile secretion, prolactin signaling pathway, sphingolipid signaling pathway, breast cancer, alcoholism, prostate cancer and cocaine addiction (Figure 5C; Supplementary Table S8). Following adjustment for multiple testing, two pathways remained significant (FDR < 0.05). Heatmaps of the metabolites in the bile secretion and prolactin signaling pathway were presented in Figures 5D,E, respectively. Enriched metabolites in the DF group sows included lithocholic acid, chenodeoxycholate, cholic acid, glycochenodeoxycholate, and progesterone (Figures 5D,E). Notably, bile secretion was the most significantly altered pathway, with subsequent findings revealing that a series of bile acids (BAs), including HDCA, CDCA, GCDCA, CA, LCA, UDCA, and γ-MCA, were significantly increased in the DF group, whereas TUDCA was significantly reduced in the DF group (Figure 5F). The results of correlation between sow reproduction performance and BAs showed that TUDCA, which was abundant in the CON group, was negatively correlated with the numbers of total born (*p <* 0.01) and healthy piglets (*p <* 0.05), GCDCA was positively correlated with the numbers of total born (*p <* 0.01), born alive (*p <* 0.05) and healthy piglets (*p <* 0.01), CDCA and HDCA were positively correlated with the numbers of total born (*p <* 0.05, Figure 5G).

### 3.6 Relationships between gut microbiota and fecal metabolites

**Figure 6:**
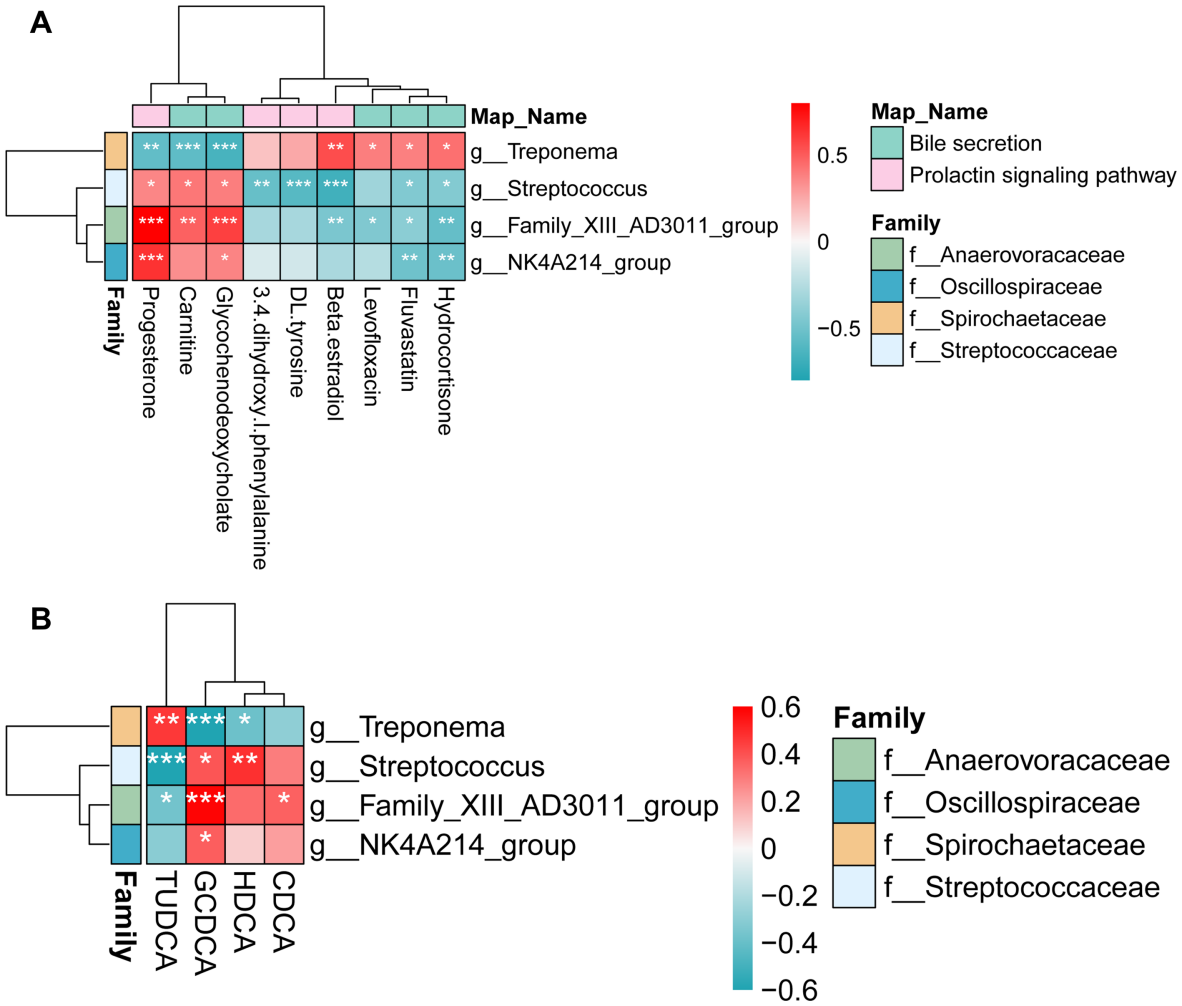
Relationship between microbiota, metabolites in differential pathways, and bile acids. **(A)** The relationship between fecal microbiota and metabolites in differential pathways. **(B)** The relationships between fecal microbiota and bile acids. Spearman’s correlation coefficients were calculated to assess the associations between bile acids and reproductive performance at G109d. Color intensity represents the correlation coefficient (ρ). *Mean a significant difference (*p* < 0.05), **mean a significant difference (*p* < 0.01), ***mean a significant difference (*p* < 0.001).

To further elucidate the potential interactions between gut microbes and metabolites associated with reproductive performance, spearman correlation analysis was conducted using the bacterial taxa and metabolites that showed significant associations with reproductive performance (Figure 6). Among these associations, particular attention was paid to *Treponema* and *NK4A214_group*, which were previously identified as key differential genera in the CON and DF groups, respectively. *Treponema* showed negative correlations with fecal progesterone (*p <* 0.01), carnitine (*p <* 0.001) and GCDCA (*p <* 0.001), but positive correlations with hydrocortisone (*p <* 0.05), fluvastatin (*p <* 0.05), levofloxacin (*p <* 0.05) and Beta-estradiol (*p <* 0.01) (Figure 6A). In addition, *Treponema* was also negatively correlated with fecal HDCA (*p <* 0.05), whereas it was positively correlated with TUDCA (*p <* 0.01) (Figure 6B). By contrast, *NK4A214_group* was negatively correlated with hydrocortisone and fluvastatin (*p <* 0.01) but positively correlated with GCDCA (*p <* 0.05) and progesterone (*p <* 0.001) (Figures 6A,B).

## 4 Discussion

The gestation period is a pivotal phase in the reproductive cycle of sows, critically influencing fetal number and development (Campos et al., 2012; Tokach et al., 2019). Nutritional intake, metabolic homeostasis, stress levels, and environmental conditions during gestation collectively shape the intrauterine milieu, thereby modulating embryonic survival rates, litter size, neonatal viability, and subsequent reproductive performance of sows (Cheng et al., 2018; Xia et al., 2021). In this study, sows fed diets containing different fiber sources exhibited significantly distinct reproductive performance outcomes. Previous study has confirmed that gestational diets with varied fiber combinations, particularly supplementation with purified guar gum and cellulose to 18.8% fiber content, significantly increased sow litter size (Zhuo et al., 2020). And when gestating diet containing high in wheat bran, wheat straw, or sugarbeet pellet meal the numbers of total born or the numbers of born alive were significantly increased (van der Peet-Schwering et al., 2003; Veum et al., 2009; Che et al., 2011). The fiber sources of the CON group included barley, wheat, wheat bran, brown rice, rice bran meal, broken rice, and sugarbeet pellet meal, whereas in the DF group, barley and brown rice were lacking, but functional fiber was supplemented additionally. The functional fiber has been studied. Supplementing an additional 2% functional fiber in the gestation diet of sows can markedly increase the birth weight of piglets and reduce the percentage of IUGR piglets (<800 g) (Wu et al., 2021).

Despite the overall improvement in total born and healthy piglets, the observed increase in white and black stillbirths in the DF group aligns with the physiological consequences of larger litter sizes in hyperprolific sows. A greater number of fetuses often results in a longer farrowing duration (Baxter et al., 2020; Peltoniemi et al., 2020), a higher risk of hypoxia for later-born piglets (Mota-Rojas et al., 2005; Choi et al., 2025) and an increased rate of stillbirths due to increased intrauterine crowding and competition for resources (Lucia et al., 2002; Chaiyapatmaetee et al., 2025). In line with this concept, the DF group showed an increase in the number of total born and a reduction in the number of IUGR piglets. This suggests that, despite a higher absolute number of stillbirths, overall fetal growth and viability were improved. Therefore, the increase in stillbirths observed may partly be due to the larger litter size and the associated peripartum challenges, such as prolonged farrowing and increased management demands (Peltoniemi et al., 2020), rather than being a direct result of the DF supplementation itself.

Given that the difference of dietary fiber and sensitivity of gut microbes influence the complex gut microbial system (Tian et al., 2020), we investigated the gut microbiome of the sows to identify critical microbiota. We found that gut microbial richness and diversity initially increased, subsequently declined, and later exhibited a recovery, which was similar to previous studies. Xu et al. (2020) reported that observed species, Chao 1 and Shannon index were higher at the late gestation stage than in early gestation and early lactation. In a Huanjiang mini-pig model, alpha diversity in jejunum and ileum gradually increased as pregnancy progresses (Xie et al., 2022). The latest study also confirmed that alpha diversity (observed ASVs) showed a significant increase or an increasing trend across different parities, from gestation day 0 to day 110 (Liu et al., 2025). Alpha and beta diversity analyses revealed that the gut microbiota structure of perinatal sows underwent significant changes, this is consistent with the findings of our previous study (Cheng et al., 2018; Xu et al., 2020). The findings in the current study revealed that gut microbiota of sows with high reproductive performance had greater stability. Alpha diversity in DF group remained stable across gestation stages, with no significant changes observed. Interestingly, during the gestation period, the significant differences in microbial diversity between the CON and DF groups were only observed at G30d. This may be because the dietary intervention in the DF group accelerated microbial remodeling, achieving a new equilibrium at G30d (Xu et al., 2024). In contrast, the CON group’s microbiota continued to undergo changes throughout gestation. Alternatively, this may be attributed to the physiological effects during early gestation are less pronounced than dietary influences, and diet has a more obvious impact on microbiota (Koren et al., 2012; Nuriel-Ohayon et al., 2016). Conversely, in late gestation and the postpartum period, the sow’s physiological state plays a more significant role in shaping microbial communities. Similarly, PC1 and PC2 analyses revealed treatment-specific temporal patterns. The DF group showed significant changes in PC1 and PC2 values between G0d and G30d; thereafter, these values remained largely stable during the remainder of the gestation period. By contrast, the CON group exhibited a progressive decline in PC1 and PC2 values throughout pregnancy. Taken together, these findings suggest that dietary supplementation with DF enhances the stability of the gut microbiota in pregnant sows, which may contribute to improved reproductive performance. The stability of gut microbiota is essential for host-microbe symbiosis, as it supports the maintenance of beneficial symbiotic bacteria and their associated functions over time (Relman, 2012; Coyte et al., 2015). The increase in microbial composition and functional diversity reflects enhanced stability and resilience of the microbial community (Tap et al., 2015; Chng et al., 2020). In our study, alpha diversity in the DF group increased during early gestation and remained stable thereafter, potentially indicating greater microbial stability in sows of the DF group.

The dominant phyla of gut microbiota in sows were highly consistent with findings from other studies (Shang et al., 2021; Li et al., 2023; Wang et al., 2025), *Firmicutes* and *Bacteroidetes* were the most dominant phyla. In fact, *Firmicutes* and *Bacteroidetes* were consistently the most abundant phyla in both groups across all periods in this study. The current study showed that the gut microbiota underwent significant changes during gestation, impacting taxonomic levels from phylum to species. A decrease in the relative abundance of *Firmicutes*, an increase in *Bacteroidetes*, and a reduced *Firmicutes*/*Bacteroidetes* ratio during gestation were linked to enhanced energy metabolism (Jumpertz et al., 2011). This suggests that sows with a lower ratio of *Firmicutes*/*Bacteroidetes* has a greater capacity to utilize dietary fiber and support host energy metabolism. Nevertheless, the findings in the current study revealed that gut microbiota of sows in CON group exhibited a greater ability to utilize dietary fiber at the late gestation, likely due to the more diverse fiber sources in the diet of CON group. Notably, the dominant genera in this study, including *Christensenellaceae_R-7_group*, *UCG-002*, *Clostridium_sensu_stricto_1*, *Rikenellaceae_RC9_gut_group*, and *Treponema*, differed to some extent from those reported in other studies on sows (Kong et al., 2016; Xu et al., 2020; Shang et al., 2021). What is more, the CON group exhibited a significant increase in the abundance of *Treponema* (Supplementary Table S4), with this genus showing a markedly upward trend from gestation day 30. According to the literatures, *Treponema*, a key hemicellulose degrader in the gut microbiota (Tokuda et al., 2018), encoded a wide array of enzymes involved in the metabolism of glucan, cellulose, arabinan, xylan, xyloglucan, chitin, pectin, and starch (Gharechahi et al., 2021). Interestingly, our results indicated that the relative abundance of *NK4A214_group* increased in DF group throughout gestation, and this relative abundance of genus at G109 d was positively correlated with the numbers of total born, born alive and healthy piglets, indicating its critical role in reproductive performance of sows. Previously classified in the family *Ruminococcaceae*, the *Oscillospiraceae NK4A214_group* has been associated with enhanced growth and lactation performance in ruminants (Tong et al., 2018; Huang et al., 2021; Wang et al., 2023). Additionally, this genus is a dominant taxon in sows (Ding et al., 2019; Wang et al., 2019; Zhuo et al., 2020; Shang et al., 2021).

To better understand how these dominant and differentially abundant genera contribute to community organization and stability, we further examined microbial interactions using genus-level co-occurrence network analysis. Thébault and Fontaine (2010) demonstrated that within mutualistic networks, higher network connectance significantly promotes community persistence and resilience. In this study, the microbial co-occurrence networks of the DF group exhibited higher connectance, density and average number of neighbors, supporting this theory. Faust and Raes (2012) further noted that high clustering coefficients, characteristic of small-world networks, contribute to maintaining the long-term stability of microbial communities. This indicates that DF intervention could improve the ecological stability of the gut microbiota by optimizing network architecture. These network-level findings are consistent with the observed diversity dynamics and ordination patterns. As shown by alpha diversity and PC1/PC2 analyses of beta diversity, the DF group experienced significant microbial restructuring during the early stages of gestation (G0–G30 d), after which the community became relatively stable. Interestingly, despite being identified as a DF-enriched taxon that is positively associated with reproductive performance, the *g__NK4A214_group* did not exhibit high centrality in the DF networks. By contrast, this genus consistently achieved high MCC scores in the CON networks at G30d and G109d, suggesting a hub-like role in the CON group. Similarly, Treponema displayed high abundance and centrality in the CON group, further indicating a centralized hub structure. In the DF group, enhanced overall network connectivity may reduce reliance on individual hub taxa, thereby distributing ecological functions across multiple interacting genera. Such decentralization is often considered a hallmark of stable and resilient microbial ecosystems (Coyte et al., 2015). Specifically, ecological network models have shown that highly centralized, single-hub networks dominated by cooperative interactions are fragile due to positive feedback loops. In contrast, shifts toward more distributed, competition-driven patterns enhance community stability by dampening these loops (Coyte et al., 2015). Our findings align closely with these theoretical predictions: the decline in the centrality of *g__NK4A214_group* in the DF group reflects a transition from a fragile, hub-dependent architecture in the CON group to a more robust, decentralized network. This transition is likely driven by DF-induced competitive dynamics that promote overall microbiome resilience.

Notably, metabolic profiling revealed significant pattern differences between the two dietary groups. Researches have shown that a fiber-rich diet can modify the composition and structure of bile acids in the host (Wu et al., 2021; Arifuzzaman et al., 2022; Hu et al., 2023), and our results indicate that bile secretion was a critical pathway in the DF group, with subsequent differential analysis of the bile acid profile further confirming this finding. A previous study showed the diet supplement with guar gum combined with pregelatinized waxy maize starch in a gestation improved bile acid homeostasis of sows which may enhance reproductive performance of sows (Wu et al., 2021). However, the bile acid profiles obtained from our non-targeted metabolomics analysis were not fully consistent with those from the targeted metabolomics analysis (Wu et al., 2021), despite both being derived from sow fecal samples collected at gestation day 109. The reason may be the differences in the diet of the control group and the distinct experimental conditions. In the current study, TUDCA was negatively correlated with the numbers of total born and healthy piglets. However, a previous study indicated TUDCA could improve intestinal barrier function and alter serum metabolites and gut microbiome in weaned piglets (Song et al., 2022). And TUDCA improved developmental competence of bovine embryos (Khatun et al., 2020), and pig embryos (Kim et al., 2012; Dicks et al., 2020), indicating its critical role in reproductive capacity. Our finding is not consistent with previous studies (Kim et al., 2012; Dicks et al., 2020), which may be attributed to several factors. On the one hand, prior studies focused on embryos, suggesting that TUDCA plays a significant role in early gestation, whereas, the samples we detected were collected from the late gestation. On the other hand, our results were derived from non-targeted metabolomics analysis, it cannot fully reflect the actual and precise situation within the sows’ bodies. We found a positive correlation between GCDCA and reproductive performance of sows, despite GCDCA is not a high concentration BA in feces, as reported by Wu et al. (2021). Previous studies have shown that GCDCA exhibits certain toxicity and can induce liver damage (Utanohara et al., 2004; Iizaka et al., 2007). Latest study suggested GCDCA or TCDCA could protect host from severe fever with thrombocytopenia syndrome infection by dampening systemic inflammatory responses in mouse model (Xie et al., 2023). However, no studies have yet established a direct link between GCDCA and reproductive capacity. A related study reported that CDCA can improve embryo implantation in sows during early gestation (Chen et al., 2024). Conjugated bile acids (CBAs), including glyco-CBA and tauro-CBA, could be hydrolyzed by bile salt hydrolase in the intestine to form free bile acids, such as cholic acid or chenodeoxycholic acid, as well as amino acids (Begley et al., 2006; Ridlon et al., 2006). This suggests that GCDCA may be able to play a role in improving reproductive performance by generating CDCA, however, whether GCDCA directly influences reproductive performance requires further investigation.

Given that several metabolites showed significant associations with reproductive performance, we next explored whether the differential bacteria identified above were correlated with these key metabolites. *NK4A214_group* was positively correlated with GCDCA and progesterone, and was negatively correlated with hydrocortisone and fluvastatin. As discussed above, GCDCA was identified as a key bile acid potentially associated with improved reproductive performance. Consistent with this observation, the current correlation analysis revealed that the *NK4A214_group*, which was positively associated with reproductive performance in the previous section, also showed a strong positive correlation with GCDCA. The convergence of these microbial and metabolic findings further supports the potential role of the *NK4A214_group* in modulating bile acid metabolism to promote reproductive performance. Moreover, *NK4A214_group* was positively correlated with progesterone, a key hormone essential for the establishment and maintenance of pregnancy. According to previous studies, plasma progesterone concentrations in sows increase rapidly after oestrus, peaking on days 12–14 of pregnancy. They then gradually decrease to a stable level that is maintained throughout gestation (Ka et al., 2018). Muro et al. (2022) reported that oral supplementation with altrenogest, a progesterone analog, from days 6 to 12 of gestation significantly increased the total number of piglets born and born alive, while reducing stillbirth rate and the proportion of low-birth-weight piglets. Collectively, these findings imply that *NK4A214_group* may contribute to improved reproductive performance through modulation of progesterone metabolism or signaling. However, as the correlation analysis in this study was based on samples collected during late gestation, further studies are needed to establish whether the *NK4A214_group* influences progesterone regulation in the early stages of pregnancy.

## 5 Limitations and future perspectives

However, the limitations of the present study should be acknowledged. Firstly, although significant associations were observed between gut microbial taxa, fecal metabolites and reproductive performance, correlation analyses cannot prove causation. Therefore, it remains to be verified whether the identified bacteria and metabolites directly mediate the beneficial effects of DF supplementation. Secondly, due to the absence of serum samples in this study, we were unable to assess systemic metabolic status or metabolite concentrations in key tissues directly. Therefore, any associations identified between fecal metabolites and whole-body metabolism or reproductive outcomes should be interpreted with caution, given that fecal profiles may not fully represent changes at the circulating or tissue level. Future studies incorporating paired serum and tissue sampling would help to validate and extend these findings. Thirdly, although significant microbial remodeling occurred in the early stages of pregnancy, this study did not investigate key events such as embryo implantation or placental development, which may represent critical periods during which dietary fiber intake can influence reproductive outcomes. Finally, the functional fiber used in this study consisted of resistant starch and guar gum. The individual contributions and potential synergistic effects of these two components were not distinguished.

Therefore, future studies should focus on mechanistic validation using approaches such as fecal microbiota transplantation or targeted bacterial supplementation, in order to establish causal links between modulation of the gut microbiota and reproductive performance. Additionally, integrating targeted metabolomics with host physiological indicators, including circulating hormones, inflammatory markers and metabolic parameters, will be crucial for elucidating the interactions between the host, microbiota and metabolites underlying the beneficial effects of functional fiber supplementation.

## 6 Conclusion

Dietary supplementation with 1% functional fiber during gestation significantly improved sow reproductive performance. These benefits were accompanied by enhanced gut microbial stability and postpartum resilience. Notably, fiber-responsive taxa, including *NK4A214_group* and members of *Family_VIII_AD3011_group* and *norank_f_Eubacterium_coprostanoligenes_group*, emerged as candidate bacteria associated with improved reproductive outcomes. DF supplementation was also associated with shifts in microbial metabolic profiles, particularly those related to bile acid and energy metabolism. Together, these findings highlight functional fiber as a promising nutritional strategy to improve sow gut health and productivity.

## Data Availability

The 16S rRNA sequencing raw data during the current study was uploaded and registered in the NCBI SRA database with accession number PRJNA1364848, https://www.ncbi.nlm.nih.gov/bioproject/PRJNA1364848. Other raw datasets may also be requested from the corresponding author provided that all ethical requirements have been met.

## Footnotes

1
https://www.bioincloud.tech
