# Supplementary material for: Maternal supplementation of functional fiber improves reproduction performance by modulating gut microbiota during pregnancy
Source: Front Microbiol. 2026 Feb 24;17:1758091. doi: 10.3389/fmicb.2026.1758091 (PMC12974138; doi:10.3389/fmicb.2026.1758091)
Supplement: Supplementary file 1 [file Data_Sheet_1.pdf]

# Supplementary information

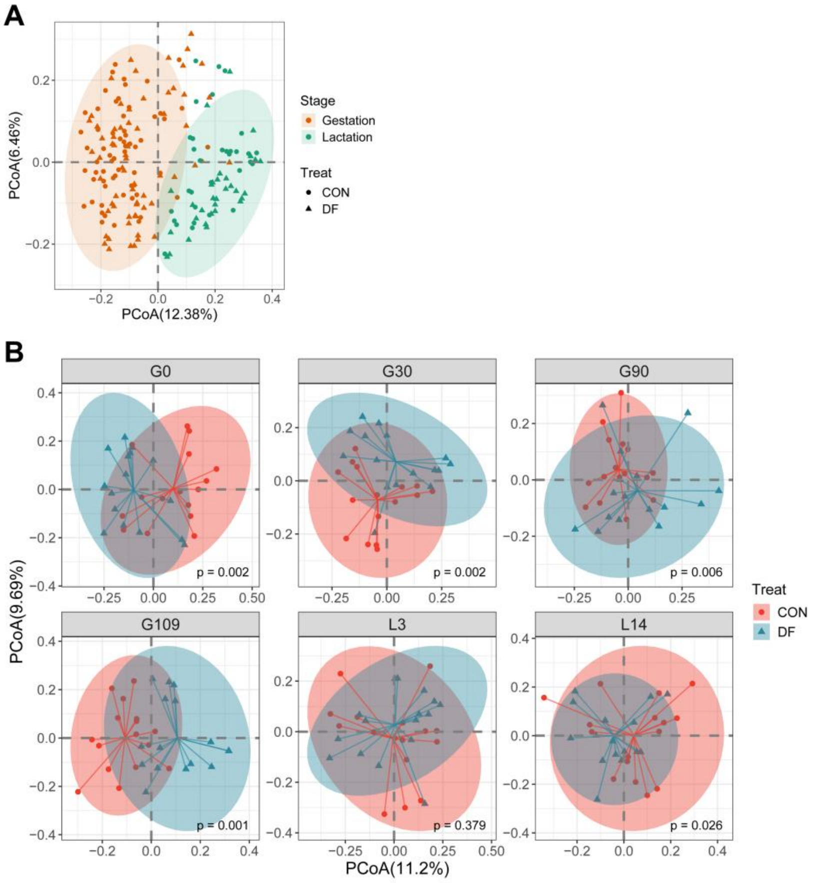


## FIG S1 Effects of different diet on fecal beta diversity in sows.

(A) Beta diversity clustered by gestational stage. (B) PCoA of the gut microbiota in sows at different gestational stages.

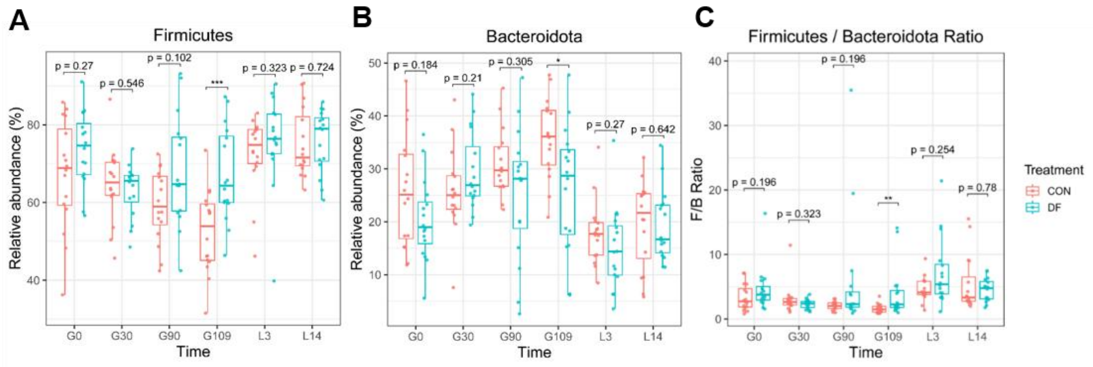


**FIG S2 Differential analysis of Firmicutes, Bacteroidota, and the Firmicutes/ Bacteroidota ratio.**

(A) Relative abundance and differential analysis of Firmicutes. (B) Relative abundance and differential analysis of Bacteroidota. (C) Differential analysis of Firmicutes/ Bacteroidota ratio. Results are expressed as median and quartile. $P < 0.05$ indicates statistical significance (*$P < 0.05$, **$P < 0.01$, ***$P < 0.001$, and ****$P < 0.0001$)

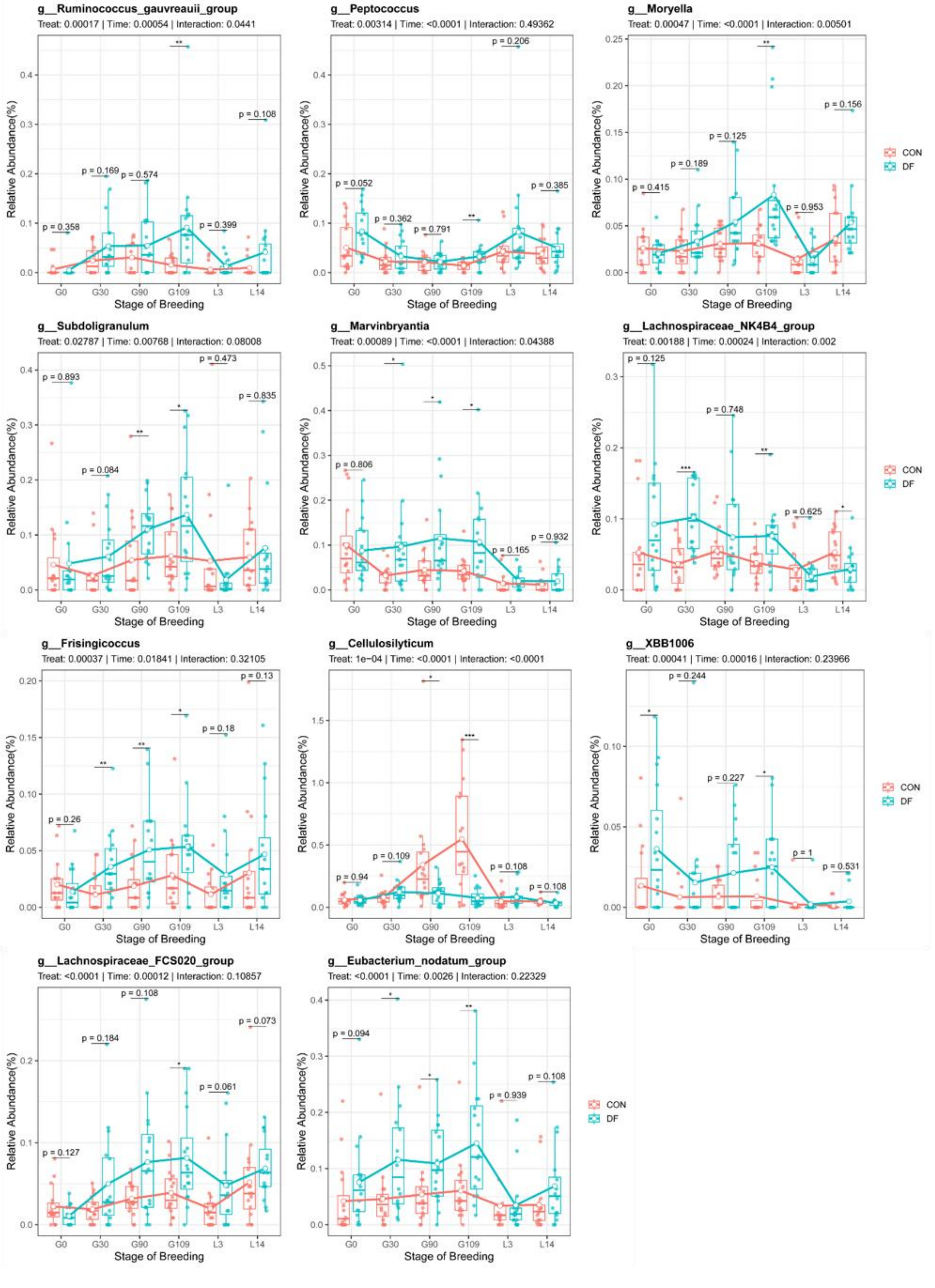


**FIG S3 Differential genera identified by both LMM and LEfSe analyses with relative abundance < 0.1%.**

Results are expressed as median and quartile. $P < 0.05$ indicates statistical significance (*$P < 0.05$, **$P < 0.01$, ***$P < 0.001$, and ****$P < 0.0001$)

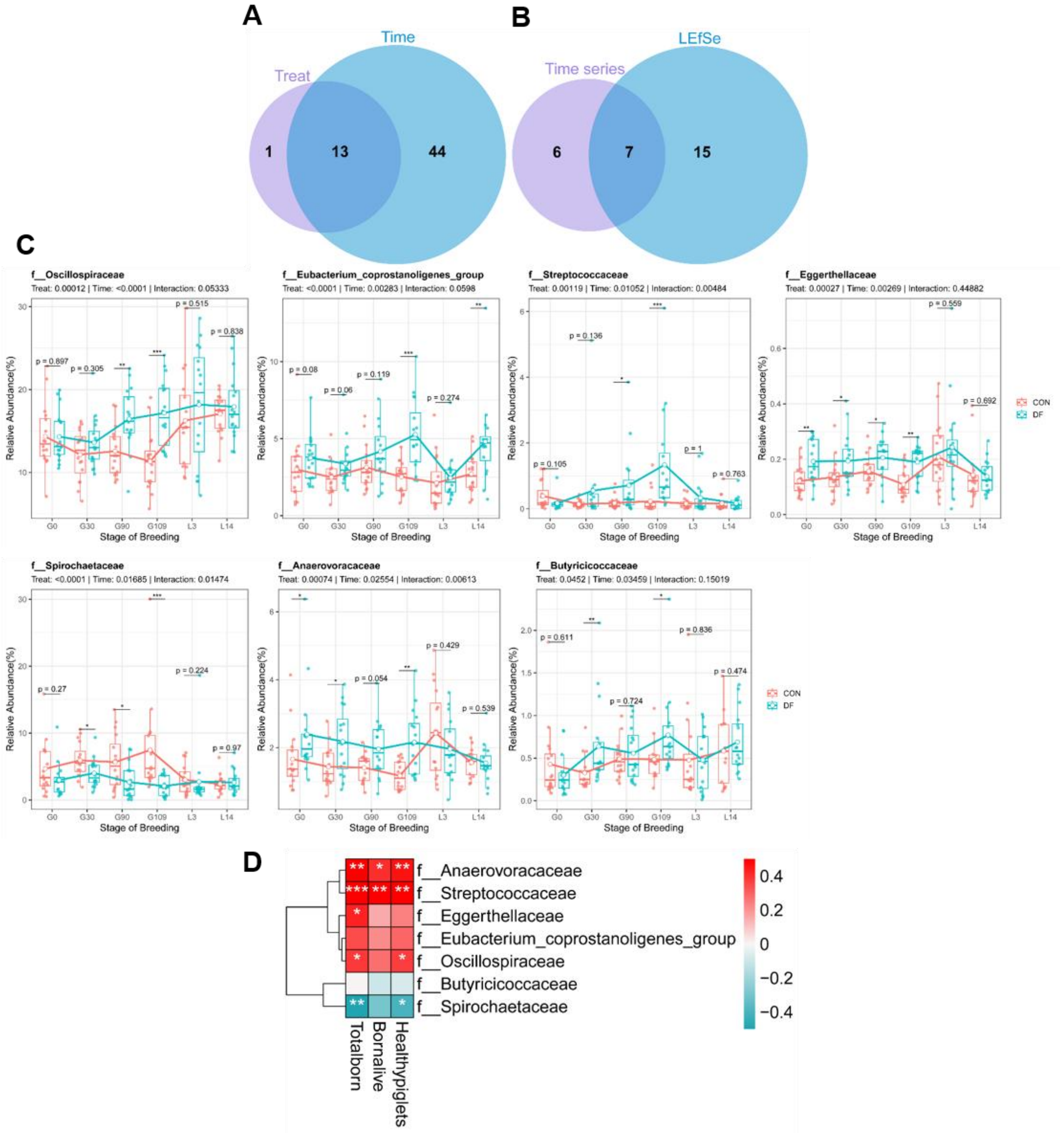


**FIG S4 Key bacterial families identified by linear mixed model (LMM) and LEfSe analyses.**

(A) The overlap between families with significant differences in treatment and stage identified by LMM, and (B) the intersection of these taxa with those identified by LEfSe analysis. (C) Differential families identified by both LMM and LEfSe analyses. (D) Relationships between the overlapping taxa identified in panel C and reproductive performance. Spearman's correlation coefficients were calculated to assess the associations between bacterial genera and reproductive performance parameters at G109d. Color intensity represents the correlation coefficient (ρ). Results are expressed as median and quartile. $P < 0.05$ indicates statistical significance (*$P < 0.05$, **$P < 0.01$, ***$P < 0.001$, and ****$P < 0.0001$).
